# PLLA Honeycomb-Like Pattern on Fluorinated Ethylene Propylene as a Substrate for Fibroblast Growth

**DOI:** 10.3390/polym12112436

**Published:** 2020-10-22

**Authors:** Klára Fajstavrová, Silvie Rimpelová, Dominik Fajstavr, Václav Švorčík, Petr Slepička

**Affiliations:** 1 Department of Solid State Engineering, University of Chemistry and Technology Prague, 166 28 Prague, Czech Republic; klara.neznalova@vscht.cz (K.F.); dominik.fajstavr@vscht.cz (D.F.); vaclav.svorcik@vscht.cz (V.Š.); 2 Department of Biochemistry and Microbiology, University of Chemistry and Technology Prague, 166 28 Prague, Czech Republic

**Keywords:** honeycomb film, surface morphology, poly(l-lactic) acid, fluorinated polymer, cell response, scaffold for cell culture

## Abstract

In this study, we present the surface patterning of a biopolymer poly(l-lactide) (PLLA) for fibroblast growth enhancement. The patterning is based on a self-organized pore arrangement directly fabricated from a ternary system of a solvent-nonsolvent biopolymer. We successfully created a porous honeycomb-like pattern (HCP) on a thermally resistant polymer—fluorinated ethylene propylene (FEP). An important preparation step for HCP is activation of the substrate in Ar plasma discharge. The polymer activation leads to changes in the surface chemistry, which corresponds to an increase in the substrate surface wettability. The aim of this study was to evaluate the influence of the PLLA concentration in solution on the surface morphology, roughness, wettability, and chemistry, and subsequently, also on fibroblast proliferation. We confirmed that the amount of PLLA in solution significantly affects the material surface properties. The pore size of the prepared layers, the surface wettability, and the surface oxygen content increased with an increasing amount of biopolymer in the coating solution. The optimal amount was 1 g of PLLA, which resulted in the highest number of cells after 6 days from seeding; however, all three biopolymer concentrations exhibited significantly better results compared to pristine FEP. The cytocompatibility tests showed that the HCP promoted the attachment of cell filopodia to the underlying substrate and, thus, significantly improved the cell–material interactions. We prepared a honeycomb biodegradable support for enhanced cell growth, so the surface properties of perfluoroethylenepropylene were significantly enhanced.

## 1. Introduction

The surface morphology and roughness of materials significantly influence cellular behavior in applications, including cell seeding [1]. In general, the cells do not prefer extremes, i.e., neither a perfectly smooth surface, nor a too rough surface. Many scientific groups have demonstrated that a patterned surface of polymer substrates provides a more attractive environment for cells than a flat surface, because it better mimics the in vivo conditions [2,3]. The advantage of a structured surface is the spatial control of cell growth. It is possible to control the final properties of prepared structures and subsequent cellular behavior by varying the parameters during material/surface preparation [4].

One of the possibilities is the formation of porous films with various pore shapes. A typical example of a porous surface inspired by nature is the formation of hexagonal units, which is called honeycomb-like patterning (HCP) [5]. Nishikawa et al. studied honeycomb films and their application as directional guidance for various cell types [6]. Several approaches to HCP preparation exist. Some techniques are more demanding stepwise (e.g., lithography) [7], and some take place under more demanding conditions (e.g., a breath figure, which requires a humid environment) [8]. Bui et al. showed how to solve these limitations and designed a uniform HCP-like film via improved phase separation, which is a simple and inexpensive method [9]. The improvement is based on the presence of methanol in a binary mixture of organic solvents. Methanol substitutes for a humid environment, stabilizes water droplets, and guarantees the creation of a regular pattern.

In tissue engineering, synthetic polymers, whose advantage is their easy formability [10], and biopolymers [11], for which there is no risk of adverse reactions in the human body, are extensively investigated. So far, the most widely used biopolymer for improving or replacing human tissues has been poly(l-lactide) (PLLA). The positive effect of PLLA as a cell scaffold has been studied for a long period [12,13]. An improved phase separation technique was also used by the application of a “base” polymer layer with a consequent dip coating technique [14]. This process (IPS) was developed for biocompatible and biodegradable PLLA, but was also tested for several other commercially available polymers, such as polymethylmethacrylate (PMMA) or polystyrene (PS) [14]. Honeycomb structures were also observed for PS using the breath figure (BF) method [15,16]. The creation of highly ordered structures was reported to only be possible under highly humid environments after drop-casting the polymer solution onto the substrate surface, as was discussed above in this text, and the preparation conditions were strictly controlled due to the inherently hydrophobic properties of the PS polymer [17]. Langmuir–Blodgett technology may also be used [18].

Our main focus of this study was to evaluate the cytocompatibility of a polymer foil when applying a PLLA HCP-like film prepared by improved phase separation. Human primary fibroblasts were chosen for this purpose, since they are widely used in various applications in clinical practice [19]. We focused on evaluating the effect of different amounts of PLLA in polymer solution on the material surface morphology, roughness, wettability, and chemistry of the prepared pattern, as well as on the response of human primary fibroblasts on the fabricated film. In our previous study, we reported on the successful design and characterization of an HCP coating from PLLA on fluorinated ethylene propylene (FEP) as a substrate, for which substrate pretreatment has been intensively examined [20]. We also tested the possibility of adding cellulose acetate to the polymer solution and the effect of the composite prepared in this way on the properties of the film [21]. The novelty of this study is the application of a flexible FEP substrate as a carrier for the construction of biodegradable and biocompatible microstructure polymer PLLA and a study of its interaction with MRC-5 cells, which has not been published so far, to the best of our knowledge. The cytocompatibility tests showed a higher number of proliferated fibroblasts on substrates coated with a PLLA porous film than on an unmodified FEP foil. The results of the analyses determined the different properties of films with different PLLA contents. The pore morphology of the prepared layer, its surface roughness, the content of elements in the surface layer, and the wettability depended on the amount of PLLA in the polymer solution. In addition, the PLLA content had a great influence on cell adhesion and proliferation.

## 2. Materials and Methods

### 2.1. Preparation of the PLLA Film

**Figure 1:**
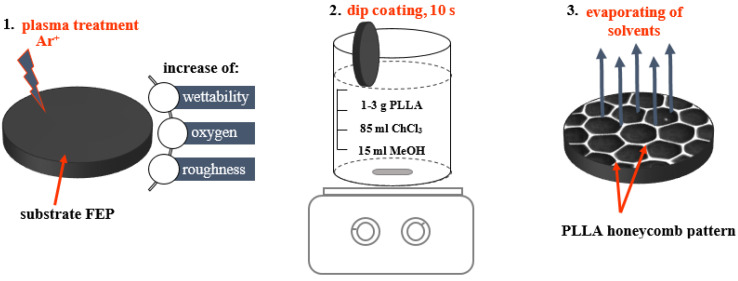
Step-by-step preparation of the poly(l-lactide) (PLLA) film.

As solvents, chloroform (CHCl_3_; stabilized with 1% ethanol A.G., M_r_ 119.38) and methanol (MeOH; for HPLC, M_r_ 32.04) obtained from Penta, Prague, CZ, were used. For HCP (coating) formation, poly-l-lactic acid (PLLA; thickness of 50 μm, oriented foil, density of 1.05 g·cm^−3^, supplied by Goodfellow Cambridge Ltd., Huntingdon, UK) was used. A circular substrate with a diameter of 20 mm, known as fluorinated ethylene propylene (FEP; a foil with the thickness of 50 μm, density of 2.15 g·cm^−3^, supplied by Goodfellow Cambridge Ltd., Huntingdon, UK), was activated by Ar plasma discharge [22] (Balzers SCD 050 device, gas purity of 99.997%, gas pressure of 10 Pa, plasma power of 8 W, exposure time of 240 s). The samples were placed on the electrode in the instrument chamber. The chamber was closed and exhausted to a pressure of 2 Pa. Ar discharge was introduced into the chamber. The substrate was attacked by Ar ions according to the set modification time, power, and working pressure. After the modification, the chamber was aerated and the samples were ready for the application of PLLA film. The modified substrate (FEP) was completely immersed in the polymer solution (100 mL; 1, 2, or 3 g PLLA, 85/15 volume ratio of CHCl_3_/MeOH) for 10 s at room temperature (RT, ca. 23 °C) and subsequently, the porous pattern was formed by evaporating both solvents (the preparation process is demonstrated in Figure 1). The improved phase separation technique is based on the different solubility of the polymer, when a “good” (chloroform in this article) and “bad” (methanol in this article) solvent is used. After removal of the substrate from the solution, fast evaporation of the chloroform took place, while methanol evaporation was much slower. This process was used as a process similar to breath figure formation, but no water vapor was applied in this study.

### 2.2. Sample Characterization

The surface morphology of the prepared PLLA structures and detailed morphology of grown fibroblasts were examined by the scanning electron microscope (SEM) using an LYRA3 device (applied acceleration voltage was 10 kV, Tescan, Brno, CZ). The samples were covered with a platinum conductive layer of a 20 nm thickness by a diode sputtering method (Quorum Q300T). The surface morphology and roughness were studied by the atomic force microscope (AFM) using a Dimension ICON device (Scan-Assyst mode, nitride lever SCANASYST-AIR, Si tip, spring constant of 0.4 N·m^−1^, Bruker Corp., Billerica, MA, USA). NanoScope Analysis software was applied for data processing. The arithmetic roughness (Ra) and root mean square (RMS) represent the average of the deviations from the center plane of the sample.

The elemental concentration was evaluated by energy-dispersive X-ray spectroscopy (EDS) using an F-MaxN analyzer and SDD detector (applied acceleration voltage was 10 kV, Oxford Instruments, Abingdon, UK). The samples were covered with a platinum conductive layer of a 20 nm thickness by a diode sputtering method (Quorum Q300T).

The wettability of the prepared PLLA structures was studied by measuring the water contact angle. The contact angle was determined by the Surface Energy Evaluation System (Advex Instruments, Brno, CZ) by applying drops of distilled water to eight different positions.

### 2.3. Fibroblast Cultivation

Human primary lung fibroblasts (MRC-5) were purchased from the American Tissue Culture Collection (ATCC, Gaithersburg, MD, USA) as a frozen vial, which was unfrozen in a water bath of 37 °C. Then, it was gently centrifuged (500× *g*, 1 min), the liquid was removed, and the cell pellet was gently resuspended with fresh prewarmed (37 °C) media. Nest, the cells were transferred to a dish with 10 mL of media. The cells were cultured according to the instructions of the manufacturer in Minimal Essential Media (MEM) supplemented with 2 mM L-Glutamine (a stable dipeptide; Sigma Aldrich, St. Louis, MO, USA) and 10% fetal bovine serum (FBS, Thermo Fisher Scientific, Waltham, MA, USA). The cells were maintained at the exponential phase of growth at 37 °C, 5% CO_2_, and 95% humidity. The cells were passaged at a subcultivation ratio of 1:4 twice a week using 0.25% (*w*/*v*) trypsin-0.53 mM EDTA solution, and were allowed to detach for ca. 5 min. at 37 °C. Then, they were gently removed by fresh complete growth media and transferred to a new dish. The experiments were performed within 16 cell divisions, so that we were sure that the cells maintained their primary phenotype, even though the manufacturer claims that the cells are capable of 42–46 population doublings before the onset of senescence. No morphological changes were observed during the experiments.

### 2.4. Cell Viability Assay

The viability of MRC-5 cells was measured using an assay that measures the cellular viability, known as the WST-1 test (Sigma Aldrich, St. Louis, MO, USA), as in ref. [23], which consists of the transformation of WST-1 reagent into colored formazan. First, the tested samples were placed at the bottoms of 12-well dishes (Ø 2.14 cm, VWR, Radnor, PA, USA), sterilized by 70% ethanol (99.9% purity) for 30 min., rinsed with sterile phosphate buffered saline (PBS, pH 7.4), and weighted to the bottom of the wells with poly(methyl)methacrylate cylinders (Zenit, Prague, CZ). Then, 15,000 cells per cm^2^ were seeded onto the samples in 1 mL of complete MEM in triplicate. After 1, 3, and 6 days of cultivation, the medium was removed, and the cells cultured on the PLLA samples and controls were rinsed with PBS, which was then replaced with WST-1 (1:20) soluted in complete phenol red-free MEM medium (Thermo Fisher Scientific, Waltham, MA, USA); the cells were incubated for 2 h. After that, the culture medium was transferred to sterile 96-well plates (100 μL per well, 4 wells per sample) and the absorbance of the arisen formazan was spectrophotometrically recorded at 450 nm (the reference wavelength of 650 nm). As controls, MRC-5 cells seeded on standard tissue culture polystyrene wells (PS) of 12-well dishes and on pristine FEP were used.

### 2.5. Preparation of Cell Samples for Scanning Electron Microscopy

In order to examine the detailed morphology of MRC-5 cells attached and growing on the tested samples, SEM (TESCAN LYRA3 GMU, Tescan, Brno, CZ) analysis in a secondary-electron mode was performed. For this purpose, the cells were seeded as described in chapter 2.4. Then, they were washed with prewarmed (37 °C) PBS and fixed with Karnovsky solution in 0.1 M cacodylate buffer (pH 7.2) for 2 h, as described in [24]. After that, they were dehydrated using an increasing percentage of ethanol in water (50%, 60%, 70%, 80%, and 90% each 10 min, two times absolute ethanol for 10 min), which was followed by two incubations (10 min each) with hexamethyldisilazane (Sigma Aldrich, St. Louis, MA, USA) and a final drying step in an oven at 30 °C for 8 h. The dehydrated samples were then coated with a ca. 20-nm platinum layer and subjected to microscopy.

## 3. Results and Discussion

Based on the principle of improved phase separation, we were able to create a polymer HCP on a modified polymer foil. We discussed the beneficial effect of plasma modification on the FEP polymer, especially for improvement of the surface wettability, chemistry, roughness, and cytocompatibility enhancement, in our previous study [22]. The contact angle of non-modified FEP was determined to be 103.5° [22].

### 3.1. Surface Morphology–SEM Analysis

**Figure 2:**
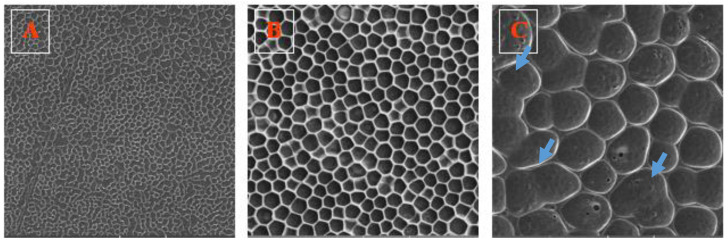
The images (100 × 100 µm^2^) obtained from scanning electron microscopy (SEM) of fluorinated ethylene propylene (FEP) foils modified at 8 W and 240 s, and then immersed into polymer solution containing (**A**) 1 g, (**B**) 2 g, and (**C**) 3 g of PLLA. Missing pore walls are indicated with arrows (selected ones).

First of all, we were interested in the effect of the PLLA content in the coating solution under otherwise constant conditions (solvent volume ratio and plasma modification parameters). The surface morphology of the HCP prepared with different amounts of PLLA (1, 2, and 3 g) is shown in Figure 2. We can observe that the higher the PLLA content in the solution, the larger the pores that were formed (Figure 2C). In ref. [9], Bui et al. reported a slighty opposite trend. This discrepancy can be explained by the fact that, although the conditions of the experiment were very similar, they differ in the use of the substrate. Bui et al. applied a layer of PLLA on a glass substrate, whereas we used a fluorinated polymer which was plasma-activated. We previously investigated the influence of plasma modification parameters on the formation of HCP-like structures from PLLA [20]. At a lower power (3 W) and different plasma exposure times, the pore size was the same; on the contrary, at a higher power (8 W), the pore size changed at different modification times. The influence of the substrate and its plasma modification reduce the pore size due to the increased surface free energy (wettability), where the higher free energy probably leads to the “lower sticking” of PLLA macromolecular material (PLLA). Therefore, its lower amount on the surface during pattern formation influences the pore size. The biopolymer macromolecules from the PLLA solution interact with the perfluorinated substrate. If the substrate is more wettable (higher degree of plasma modification and presence of oxygen on the surface), the biopolymer macromolecules are easily attracted to the surface during the process of honeycomb formation. Considering pristine FEP, also due to its hydrophobic character, the PLLA solution (and biopolymer macromolecules) does not have a sufficient interaction time, so the pattern is not created. The pore size increases with the PLLA amount in the solution, since the improved phase separation is strongly affected by the amount of source biopolymer in the solution, thus affecting the PLLA pore size.

**Figure 3:**
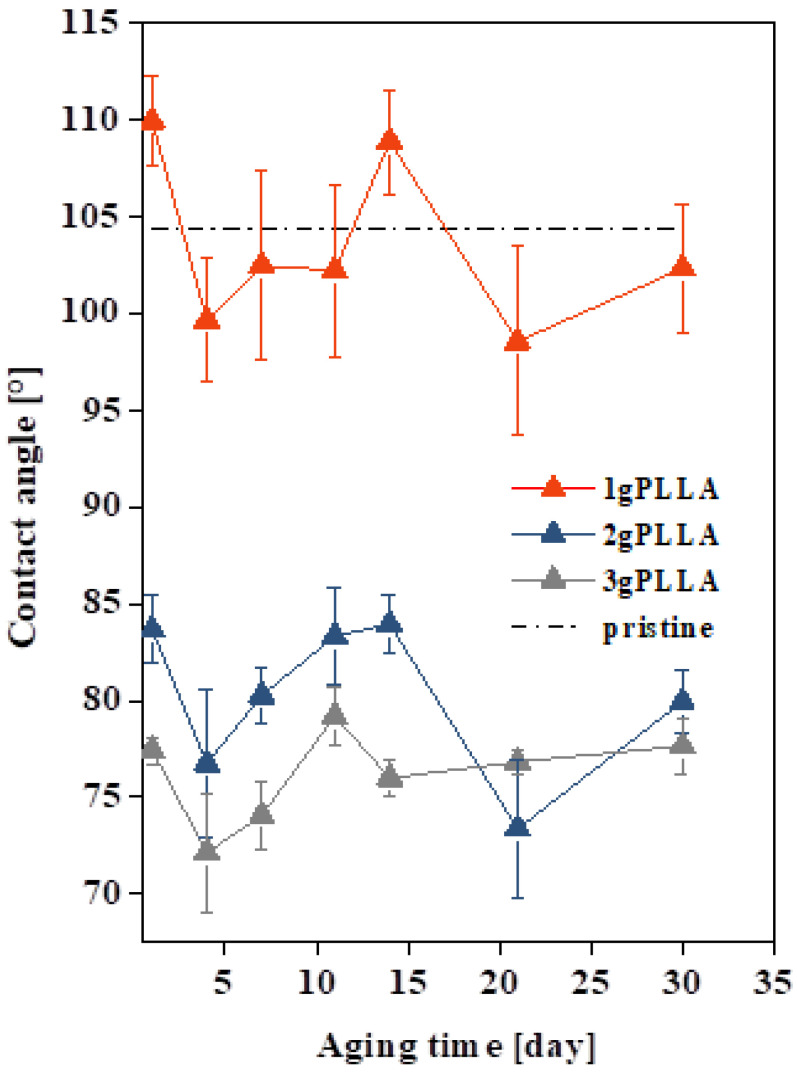
The contact angles and aging of unmodified FEP (pristine) and FEP foils modified at 8 W and 240 s and then immersed in polymer solution containing 1, 2, or 3 g of PLLA.

We can conclude that the conditions during plasma pretreatment have a great influence on the resulting HCP. In this experiment, we worked with the parameters that showed the most promising results, according to our previous measurements (8 W and 240 s). Moreover, the shape of the pores was the most regular in a layer containing 2 g of PLLA (Figure 2B), with a pore diameter of approx. 5 µm; however, some variations in the pore sizes may be found. On the contrary, the layers with 1 g (Figure 2A) and 3 g of PLLA (Figure 2C) did not have periodic pores of similar parameters and in some places, missing pore walls were visible. Therefore, not only plasma modification, but also the PLLA content, had a great influence on the layer morphology.

### 3.2. Surface Wettability

Furthermore, we also measured the wettability of the presented porous layers, depending on the amount of PLLA in the polymer solution for one month (Figure 3). In the graph, the dashed line shows the value (104.4°) of the contact angle of the unmodified substrate (pristine). The influence of plasma parameters on the prepared pattern and corresponding changes in the contact angles were investigated previously [20]. For samples with 2 and 3 g of PLLA, the contact angles decreased immediately after the first day of aging in comparison to pristine FEP sample, which corresponds to a sharp increase in the surface wettability. One can also notice that the layer with 1 g of PLLA had larger deviations and the values of the contact angles were similar to those of the pristine FEP sample. This may have been caused by an inhomogeneous and discontinuous polymer coating. Between the curves with 1 and 2 g of PLLA, there is a rapid difference in the value of the contact angle. With a higher PLLA content, the contact angle dropped from 110° to ca. 85°. It is known that a higher oxygen content in surface layers leads to a surface wettability [25] increase. As will also be discussed further, the increase of oxygen is caused by two main factors: By PLLA microstructure formation (for 2 and 3 g PLLA) and a combination of the former factor and remaining oxygen on the activated FEP substrate. As is obvious from Figure 3, the key factor for the wettability increase for layers prepared from 2 and 3 g PLLA solution is the presence of oxygen from the PLLA microstructure. However, a minor role in wettability is also played by the pattern morphology, even though it is commonly known that a significant effect of surface morphology on wettability is observed for larger structures than we prepared; however, the dependence is very complicated [26]. A different situation was observed for 1 g PLLA (Figure 3). One can expect lower contact angles (combination of surface oxygen from PLLA and FEP), but the inhomogeneous PLLA consisting of a network with in-between areas of FEP led to an increase of the contact angle, which was higher than for the samples with a continuous PLLA layer. The PLLA network morphology of a low height was probably responsible for the contact angle increase for 1 g PLLA.

Usually, during the aging of the plasma-exposed polymer, the wettability of the surface decreases after a few days (the polarity of the surface decreases). This is due to the reorientation of oxygen functional groups formed during plasma pretreatment towards the volume of the material. In our case, we did not observe this effect, due to the character of the surface (biopolymer microstructures on plasma-exposed fluoropolymer). The fluctuating values of the contact angles of all samples during the whole month can probably be justified by the plasma modification of the substrate before the application of the layer. During the aging of the substrate, chemical changes occurred in the surface of the polymer, which led to a change in the wettability [27]. Over the course of 30 days, one can observe a similar trend for all three curves, with the surface wettability increasing in the following order: 1 g < 2 g < 3 g of PLLA.

### 3.3. Surface Chemistry

**Figure 4:**
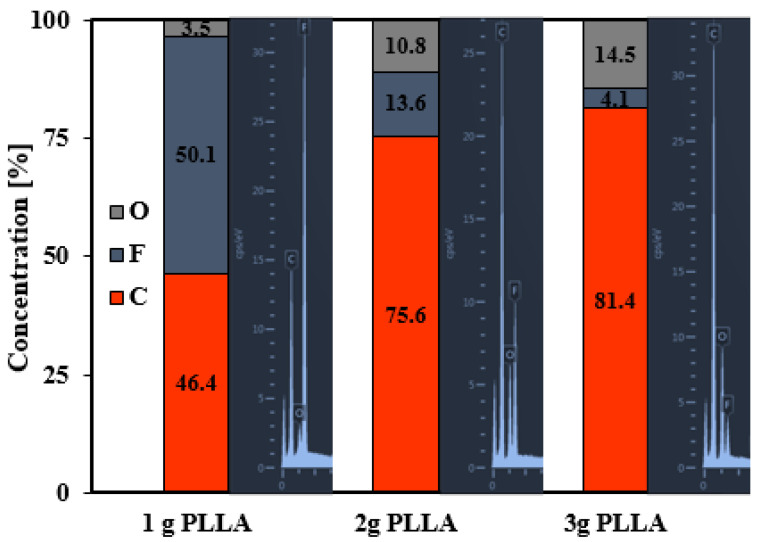
The surface concentration of elements (C, F, and O) on FEP foils modified at 8 W and 240 s and then immersed in polymer solution containing 1, 2, or 3 g of PLLA. The diagrams are presented with energy-dispersive X-ray spectroscopy (EDS) elemental signals.

As well as the wettability, surface chemical changes depending on plasma treatment were studied in the previous study [20]. This time, we focused on changes in surface chemistry, depending on the amount of PLLA in the HCP-like layer (Figure 4). The expected increase in oxygen occurring with an increasing PLLA concentration in the coated solution is evident. A higher amount of oxygen was present due to the partial replacement of fluorine by oxygen after plasma modification and, at the same time, a higher amount of PLLA, in which the oxygen is part of the polymer chain, unlike in the FEP substrate. As aforementioned, the surface chemical composition is closely related to the surface wettability. The results of EDS correspond to the results of goniometry measurement, for which we obtained a more wettable surface with an increased oxygen content.

### 3.4. Surface Roughness, Morphology and Surface Area–AFM Analysis

**Figure 5:**
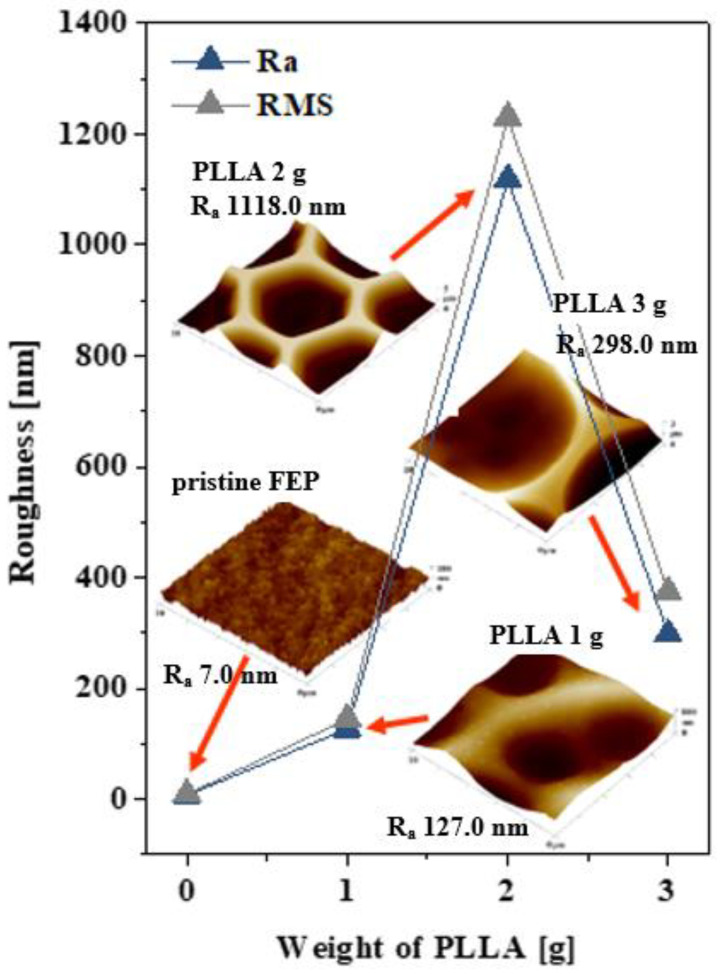
The surface roughness (R_a_, RMS) and corresponding images obtained from atomic force microscopy (AFM) of unmodified FEP (pristine) and FEP foils modified at 8 W and 240 s, and then immersed in polymer solution containing 1, 2, or 3 g of PLLA. AFM images of 10 × 10 μm^2^ were introduced.

A graph of the dependence of roughness (R_a_ and RMS) on the amount of PLLA supplemented with the corresponding images of the surface morphology obtained from AFM is introduced in Figure 5. The sample with 2 g of PLLA surprisingly exhibited a large increase in roughness (1118.0 nm). From the AFM images, we can observe that, at 1 g of PLLA, the pore walls were the thickest, whilst increasing the PLLA narrowed them, and it looked as if the bottom of the pores was also covered with PLLA. The roughest surface had a layer with 2 g of PLLA. In contrast, pristine FEP demonstrated low values of average roughness (Ra) (7.0 nm) and root mean square roughness (RMS) and we can observe a wrinkled pattern on the surface. Images obtained from AFM confirmed the results from SEM analysis, where the lateral pore size increased with an increasing amount of PLLA. The surface areas for the studied samples were as follows: for pristine FEP, the surface area (Sa) was 100 μm^2^; PLLA 1 g, the Sa was 102 μm^2^; PLLA 2 g, the Sa was 239 μm^2^; and PLLA 3 g, the Sa was 110 μm^2^. Bui reported three main points regarding an appropriate choice of methanol as a nonsolvent to induce phase separation in normal air and to support the formation of an ordered structure. Firstly, its high affinity with water. Therefore, the enrichment of methanol in the solution through solvent evaporation is favorable for absorbing water vapor from the ambient environment. Secondly, the high solubility with water-immiscible solvents, such as chloroform, was reported, and last but not least, methanol accumulation was reported to induce the transport of polymers to the surface of nonsolvent droplets [9]. It was also reported that the pore sizes of the honeycomb films can be further tuned by modulating the ambient humidity and polymer concentration [9]. One of the results presented in [9] was that the pore sizes slightly decreased with an increasing concentration of the biopolymer in the solution. Our findings regarding the influence of the amount of methanol are in good agreement with Bui [9], and our preliminary testing of an increasing humidity also confirmed the results reported in the experiment of [9]. Our experiments presented in this article were performed under a constant humidity and ambient atmosphere. As Bui reported for the PLLA/glass system, a higher polymer concentration should provide a larger viscosity, thus leading to the formation of slightly small pores. Our results indicate that, with an increasing amount of PLLA, the pore size increased. This phenomenon is probably based on different surface wettability and roughness values, which also significantly change the surface entropy, since entropy was also discussed in [9], where the slightly increased entropy was reported to be responsible for the slightly smaller pore diameter. Plasma activation for honeycomb pattern formation was also successfully applied by Babyal et al. [28] under a controlled humidity. The fluorine plasma led to the formation of superhydrophobic surfaces.

### 3.5. Cell Viability

**Figure 6:**
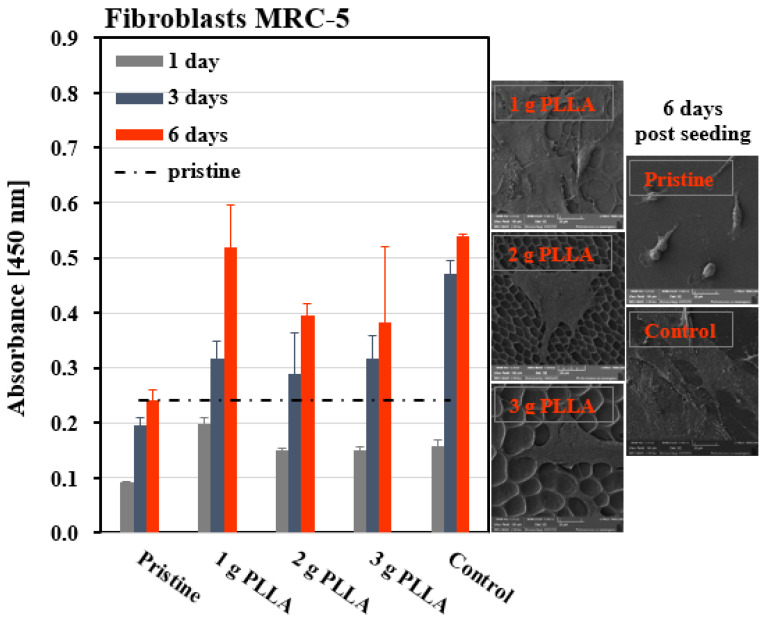
Viability of human primary lung fibroblasts (MRC-5) and corresponding images (100 × 100 µm^2^) obtained from scanning electron microscopy (SEM) of unmodified FEP (Pristine); standard tissue polystyrene (Control); and FEP foils modified at 8 W and 240 s and then immersed in polymer solution containing 1, 2, or 3 g of PLLA.

The second step of our work was to monitor the behavior of human primary fibroblasts (MRC-5) on the porous pattern. All prepared samples were evaluated for MRC-5 viability using the WST-1 assay 1, 3, and 6 days after seeding (Figure 6). As controls, pristine FEP (Pristine) and standard tissue culture polystyrene (Control) were used. If we compare the viability of fibroblasts growing on samples with PLLA, the cells on a sample with 1 g of PLLA, i.e., the sample with the smallest pores, performed the best during the whole cell cultivation period. The number of cells was comparable to the control on day 6. The reasons for this are probably the size of the pores and the discontinuity of the layer. In the previous article, we studied the cytocompatibility of the plasma-modified FEP substrate and the results indicated an increased cytocompatibility of FEP after plasma exposure. Plasma pretreatment of the FEP substrate, which leads to different surface morphologies, not only affects the number of adhered cells, but also their shape [22]. Therefore, fibroblasts may have performed even better at places on the polymer surface, on which the plasma-modified substrate was not coated with the PLLA micropattern—HCP. The cells grew to a greater extent on all substrates than on the pristine sample during the whole culture period. SEM images were added to the graph to visualize the cell coverage of the samples and the interaction of individual fibroblasts with the underlying substrate (Figure 6). The study of the influence of the surface chemistry and morphology on the cell viability was also presented, e.g., by Sequeira et al. [29]. On the pristine FEP, the cells had a round shape, without observable actin protrusions. The images correspond to the results of the WST-1 assay, where, on both the control and the sample with 1 g of PLLA, MRC-5 cells covered almost the entire surface. In contrast, for 2 and 3 g of PLLA, the cells only partially covered the surface in some areas. In conclusion, we can state that, in comparison to the unmodified FEP, the porous structure of HCP is attractive for this type of cell. The cells had the correct physiological shape. The highest cell number was detected on the PLLA layer prepared from 1 g of PLLA solution.

## 4. Conclusions

In this study, we covered the FEP substrate with a biopolymer porous pattern for cytocompatibility improvement. The application of the PLLA layer had a positive effect on the surface properties of the substrate, and the number of cells was significantly increased on the biopolymer microstructure compared to pristine FEP. We wanted to determine the cell response on the biopolymer micro pattern with the advantage of further biopolymer support and biodegradability, and we succeeded. The wettability and roughness of the substrate increased rapidly. The cytocompatibility tests showed very positive results after the cultivation of human primary fibroblasts on the coated FEP. The effect of the amount of PLLA proved to be a very important factor for the formation of the layer with different properties considering the morphology and wettability, and the presence of PLLA microstructures proved to be a very important factor for the growth of the MRC-5 cells. The polymer solution with a higher amount of PLLA led to a layer with the largest pores and the highest surface wettability corresponding to the highest oxygen content. The highest cell number was detected on the PLLA layer prepared from 1 g of PLLA in solution (except for tissue polystyrene). From this study, we can say that the HCP pattern from PLLA can be described as comparable and non-toxic compared to tissue polystyrene. The PLLA pattern present on the treated FEP foil can be used for the enhancement of MRC-5 cell growth. For all three concentrations of PLLA, the results for the support of cell growth were significantly better when compared to pristine FEP. The higher amount of PLLA (3 g) led to a slightly more inhomogeneous distribution of cells, which is represented by the higher error of measurement. On the contrary, for a lower PLLA concentration (1 g), the number of cells was even comparable to tissue polystyrene and the cell distribution was more homogeneous compared to 3 g of PLLA. These findings could provide a new valuable direction for further research and development in the field of tissue engineering and cytocompatibility enhancement.
